# Early and long-term outcome of elective stenting of the infarct-related artery in patients with viability in the infarct-area: Rationale and design of the Viability-guided Angioplasty after acute Myocardial Infarction-trial (The VIAMI-trial)

**DOI:** 10.1186/1468-6708-5-11

**Published:** 2004-11-11

**Authors:** Ramon B van Loon, Gerrit Veen, Otto Kamp, Jean GF Bronzwaer, Cees A Visser, Frans C Visser

**Affiliations:** 1 Department of Cardiology, VU University Medical Center, De Boelelaan 1117, 1081 HV Amsterdam, The Netherlands

## Abstract

**Background:**

Although percutaneous coronary intervention (PCI) is becoming the standard therapy in ST-segment elevation myocardial infarction (STEMI), to date most patients, even in developed countries, are reperfused with intravenous thrombolysis or do not receive a reperfusion therapy at all. In the post-lysis period these patients are at high risk for recurrent ischemic events. Early identification of these patients is mandatory as this subgroup could possibly benefit from an angioplasty of the infarct-related artery.

Since viability seems to be related to ischemic adverse events, we initiated a clinical trial to investigate the benefits of PCI with stenting of the infarct-related artery in patients with viability detected early after acute myocardial infarction.

**Methods:**

The VIAMI-study is designed as a prospective, multicenter, randomized, controlled clinical trial. Patients who are hospitalized with an acute myocardial infarction and who did not have primary or rescue PCI, undergo viability testing by low-dose dobutamine echocardiography (LDDE) within 3 days of admission. Consequently, patients with demonstrated viability are randomized to an invasive or conservative strategy. In the invasive strategy patients undergo coronary angiography with the intention to perform PCI with stenting of the infarct-related coronary artery and concomitant use of abciximab. In the conservative group an ischemia-guided approach is adopted (standard optimal care).

The primary end point is the composite of death from any cause, reinfarction and unstable angina during a follow-up period of three years.

**Conclusion:**

The primary objective of the VIAMI-trial is to demonstrate that angioplasty of the infarct-related coronary artery with stenting and concomitant use of abciximab results in a clinically important risk reduction of future cardiac events in patients with viability in the infarct-area, detected early after myocardial infarction.

## Background

Management of acute myocardial infarction (AMI) has underwent considerable changes in the last two decades and the management of patients with AMI has become more established [1,2]. In patients with ST-segment elevation myocardial infarction (STEMI), primary angioplasty is becoming first-choice therapy [2-4]. However, because of the low availability of such treatment, even in developed countries, most patients with STEMI are reperfused with intravenous thrombolysis. Many patients do not receive a reperfusion therapy at all [5,6].

Data from large clinical trials indicate that after successful thrombolysis more than 50% of patients have a significant residual stenosis and about 30% of patients have spontaneous or inducible ischemia [7-9]. In this group reocclusion of the infarct-related artery is a potential threat, as it is associated with recurrent ischemia or recurrent infarction [10,11]. Therefore, early risk assessment is of great importance, especially in patients treated with thrombolysis and patients who did not receive reperfusion therapy. This risk assessment should be followed by an effective treatment strategy in order to prevent recurrent cardiac events and deterioration of left ventricular function [12,13]. As these recurrent events are mainly due to the presence of an unstable residual stenosis of the infarct-related coronary artery, an effective therapy should include an invasive procedure like angioplasty to optimize coronary flow to the infarct area in high risk patients [11].

Trials have been performed to study the effect of routine angioplasty on clinical outcome early after AMI treated with thrombolysis [14-20]. Most of these studies failed to show improvement of clinical outcome with a standard invasive approach. Possible explanations for these results are the unselected approach, and the high risk profile of balloon angioplasty in the early days, when abciximab and stents were not available. Because angioplasty always carries an inherent risk, it remains important to select those patients after a recent myocardial infarction, who will actually benefit from angioplasty of the infarct-related artery.

To date, non-invasive risk stratification after AMI has mainly focused on exercise stress testing. The inability to exercise, the low diagnostic accuracy and resting ECG abnormalities, however, remain important limitations in the detection of ischemia [21].

Several post-infarction observational studies investigated viability as prognosticator and showed that the presence of viability in the infarct area was highly predictive for future coronary events like recurrent ischemia, recurrent infarction, left ventricular failure, and death [22-31]. Viable tissue is potentially jeopardized by an unstable residual stenosis in the infarct-related coronary artery. In a meta-analysis of non-randomized data by Iskandrian, the impact of revascularization on clinical outcome in patients with viability after AMI was studied [32]. A significant decrease in future cardiac events was observed in the patients with viability who were revascularized. In contrast, the outcome in patients without viability in the infarct area did not change by an invasive strategy.

In this context it should be noted that other post-infarction studies have shown viability to be associated with an improved prognosis [33-36]. Two studies demonstrated that especially patients with severe LV dysfunction and viability show a better survival than patients with LV dysfunction but without viability [34,36]. The reason for this paradox is not quite understood. However, it can be argued that patients with viability have a potential of recovery of LV function (spontaneous or by revascularization), thereby improving their survival [37]. Thus, on one hand viability may improve survival by recovery of function especially in patients with moderate to severe LV dysfunction, on the other hand viability is related to a worse prognosis by increased risk of recurrent ischemic events.

Based on the aforementioned assumptions, we initiated a clinical trial to investigate the benefits of percutaneous coronary intervention (PCI) of the infarct-related artery in patients with viability detected in the early, subacute phase of myocardial infarction. To demonstrate viability, we use low-dose dobutamine echocardiography (LDDE). This test can safely be performed 48 hours after acute MI [23,27,30,31]. It is a well validated bedside test with a diagnostic accuracy of about 80%, which is comparable to scintigraphical techniques (SPECT/PET) [38]. Coronary stenting will be performed with concomitant use of abciximab. After stenting, oral clopidogrel is given in a standard way. With this approach the lowest possible periprocedural event rate will be attained, with a low rate of target vessel revascularization (TVR) [39-43].

We hypothesize that, in order to prevent future cardiac events, PCI is only useful in patients with viability in the infarct zone early after myocardial infarction.

## Methods

### Patient Selection

Patients admitted to any of the participating centers with an acute or recent myocardial infarction, who are not treated by direct or rescue angioplasty, and who are stable during the first 48 hours after the acute event, are screened for the study. Patients < 80 years of age are considered suitable for the study when they have definite myocardial infarction, as demonstrated by an significant rise in creatine kinase-MB levels (twice the upper limit of normal: ULN), 1 mm ST segment elevation in two or more standard leads or 2 mm ST segment elevation in two contiguous chest leads, and/or the development of Q waves.

The criteria for exclusion are: viability testing technically not possible (poor echo-window), contraindications for dobutamine echocardiography (arrhythmia), and coronary angiography (severe diabetic nephropathy or known contrast-allergy), serious life-threatening non-cardiac illness, ECG abnormalities making the evaluation of the ST segment impossible (left bundle branch block, pacemaker), and an unreliable follow-up.

The study complies with the Declaration of Helsinki and all ethics committees of the participating centers have approved the protocol. All eligible patients provide written informed consent.

### Study design

**Figure 1:**
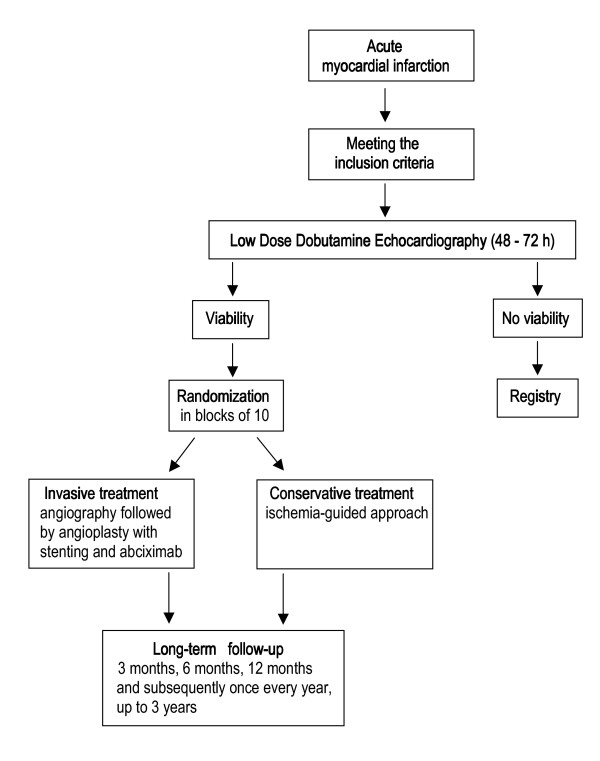
Flow chart

The study is a prospective, multicenter, randomized, controlled clinical trial. In the VIAMI-trial, patients who are admitted to the hospital with an acute myocardial infarction and who did not undergo primary or rescue PCI, are evaluated by LDDE within 3 days of admission. Patients with unequivocal signs of viability in the infarct-area are randomized to an invasive or a conservative treatment strategy. In the invasive strategy patients undergo coronary angiography with the intention to perform PCI with stenting of the infarct-related coronary artery. In the conservative group an ischemia-guided approach is adopted with stress testing before discharge from the hospital. When the test is highly suggestive for ischemia, coronary angiography will be performed. If revascularization is performed, this will be scored as a secondary endpoint. Patients without viability will serve as a registry group with long-term follow-up (Fig 1). These patients are assigned to the conservative group in order to prevent physicians' bias during the trial.

The primary endpoint is the composite of death from any cause, recurrent infarction, and unstable angina. The secondary endpoints are need for revascularization, the occurrence of angina pectoris (CCS classification), and the incidence of heart failure (NYHA classification). Left ventricular function is also evaluated as determined by echocardiography at 3 months, 6 months, and 1 year follow-up.

A reinfarction is diagnosed if there is an increase in the total creatine kinase and MB isoenzyme activity (2 times ULN) and either a history of ischemic chest discomfort, or electrocardiographic changes indicative for transmural ischemia or necrosis.

For the diagnosis of unstable angina, the patient must be hospitalized with ischemic chest pain or discomfort occurring at rest or with minimal exertion. In addition, the need for intravenous medical intervention and/or objective evidence of myocardial ischemia is required. For extensive description of the end points definitions, see Table I.

**Table I:** Primary end point definitions

**Definition of reinfarction**
1. Reinfarction during hospitalization for index infarct and not related to revascularization procedures - Either ischemic type of chest discomfort or new electrocardiographic changes indicative for transmural ischemia or necrosis with an increase in the total creatine kinase and MB isoenzyme activity. The activity of CK-MB has to be at least 2 times the upper limit of normal and more than 50% above the previous baseline value.
2. Reinfarction discharge for index infarct and not related to revascularization procedures - Either a history of ischemic chest discomfort, usually lasting > 20 minutes, or classic electrocardiographic changes indicative for transmural ischemia with an increase in the total creatine kinase and MB isoenzyme activity of at least 2 times the upper limit of normal. - New abnormal Q-waves (amplitude ≥ 1/3 of total QRS amplitude and ≥ 0.04 seconds) in ≥ 2 contiguous leads.
3. Periprocedural reinfarction during revascularization after index infarct PCI - Classic electrocardiographic changes indicative for transmural ischemia with an increase in the total creatine kinase and MB isoenzyme activity of at least 2 times the upper limit of normal. - New abnormal Q-waves (amplitude ≥ 1/3 of total QRS amplitude and ≥ 0.04 seconds) in ≥ 2 contiguous leads.
CABG
- Classic electrocardiographic changes indicative for transmural ischemia with an increase in the total creatine kinase and MB isoenzyme activity of at least 5 times the upper limit of normal. - New abnormal Q-waves (amplitude ≥ 1/3 of total QRS amplitude and ≥ 0.04 seconds) in ≥ 2 contiguous leads.
**Definition of unstable angina**
- Ischemic type of chest discomfort at rest or with minimal exertion, with a duration of at least 15 minutes. The presenting symptoms must represent a change from the patients' usual anginal pattern. - Either a need for intravenous medical intervention and/or objective evidence of myocardial ischemia (dynamic ST changes in ≥ 2 contiguous electrocardiographic leads, or an abnormal elevation of Troponin-T or Troponin-I without a significant rise of CK MB isoenzyme activity)

PCI, percutaneous coronary intervention; CABG, coronary artery bypass grafting; CK-MB, creatine kinase myocardial enzyme

A clinical endpoints and safety monitoring committee will continuously check and monitor all events in a blinded manner. Reinfarction after revascularization will be scored as described in Table I. Major and minor bleeding complication in the invasive treated group are defined according to the criteria of the Thrombolysis in Myocardial Infarction trials [44]. All patients will be treated with aspirine, beta blockers, angiotensin-converting-enzyme inhibitors and statins as accepted in international guidelines [2,4].

### Low-dose dobutamine echocardiography

If the inclusion criteria are met and none of the criteria for exclusion, viability testing by LDDE is performed within 3 days of admission.

Preferably, beta-blockers are withdrawn 24 hours before the test [23,27,30,31]. Discontinuation of beta-blockers seems to to enhance LDDE accuracy [45,46]. Before the administration of dobutamine, a baseline echocardiogram is obtained according to the guidelines of the American Society of Echocardiography [47]. Five standard views are obtained: the parasternal long-axis and short-axis view and the apical two, three- and four-chamber view. A 16-segment model is used in which the apex is divided in 4 segments. Segmental wall motion and thickening is scored according to a 4-point scale: 1 = normal, 2 = hypokinetic, 3 = akinetic, and 4 = dyskinetic. Left ventricular volumes and ejection fraction are measured by use of the modified Simpson's rule algorithm from orthogonal apical long-axis projections as recommended by the American Society of Echocardiography.

Dobutamine is administrated intravenously at doses of 5, 10, and 15 μg/kg/min, for 5 minutes at each dose. When a 10% increase in heart rate is not achieved with 15 μg/kg/min, a 5-minute infusion with 20 μg/kg/min can be used as the final stage of the procedure.

Viability is defined as the improvement of wall motion abnormalities in two or more segments of the infarct zone. Changes from hypokinesia to normokinesia and from dyskinesia or akinesia to hypo- or normokinesia are considered an improvement in wall motion abnormality. Dyskinesia changing to akinesia is not considered as an improvement. Patients are continuously monitored by a 12 lead ECG and blood pressure is recorded at the end of each stage. All views are recorded at rest and during dobutamine on VHS videotape. All videotapes are sent to the core-lab (VU University medical center) and will be analyzed by 2 experienced observers. A third observer is used in case of disagreement to reach consensus.

For subsequent off-line analysis, the echocardiographic images will be digitized from VHS videotape and transferred to a working station (Enconcert^® ^by Philips). The images will be displayed side-by-side in a quadscreen format to facilitate the comparison of images.

### Coronary angiography and angioplasty

Angiography and angioplasty will be performed as soon as possible after randomization. Coronary angiography will be performed according to standard protocols. To determine the severity of culprit lesions, quantitative coronary arteriography (QCA) will be performed to measure percent diameter stenosis, reference diameter and cross-sectional area stenosis. The degree of stenosis will be determined in the view in which the stenosis is most severe.

When feasible, PCI will be performed (with or without etc) when there is a significant (≥ 50%) stenosis or occlusion of the infarct-related coronary artery, with the intention to perform primary stenting of the infarct-related artery, with concomitant use of abciximab, according to the EPILOG protocol [39] After stenting, all patients recieve oral clopidogrel in a standard way. In case of severe 3-vessel disease or significant left main stem stenosis, where PCI is judged to be a high risk, coronary artery bypass grafting will be considered.

### Follow-up

Patients will be followed for a period of three years. Follow-up data will be obtained of all patients during visits at the outpatient clinic in the first year, and by telephone interview in the second and third year of follow-up. Left ventricular function (volumes and ejection fraction) is determined by echocardiography at 3 months, 6 months, and 1 year of follow-up.

### Statistical design

The VIAMI-trial is conducted to investigate the differences in clinical outcome between an invasive and a conservative strategy in patients with demonstrated viability in the infarct-area. The expected event rate in the viability positive group is estimated to be 35 percent. To demonstrate with a power of 80% (α = 0.05, two-sided) that PCI leads to a 50% reduction in event rate in the invasive group compared to the conservative group, 200 patients will be needed in each group. Therefore, we intend to randomize a total of 400 patients in this trial, with interim analysis after 200 included patients.

The formal stopping rules for the study are the following: If one of the treatment strategies appears significant superior at interim analysis (P ≤ 0.01), the study will be stopped.

### Statistical analysis

Baseline descriptive data will be presented as mean ± standard deviations (SD). Differences in clinical and echocardiographic variables will be assessed by unpaired Student's *t *test. Differences between proportions will be assessed by chi-square analysis; a Fisher's exact test will be used when appropriate. Event-free survival curves are computed with the Kaplan-Meier method, and the differences between these curves are tested with a Mantel-Cox log-rank test. A primary endpoint analysis is planned at 30 days, 6 months, and 1 year of follow-up. Subgroup analyses are planned to determine whether the treatment effect is more or less pronounced in certain subgroups. The data will be subgrouped by sex, age, diabetes, anterior infarction, time from onset of symptoms to treatment, and the use of thrombolytics. All analyses will be performed on an intention-to-treat basis. Also, the outcome per-protocol will be evaluated. Such an analysis seems worthwhile, as it will reflect the true influence of PCI on post-thrombolytic ischemic events.

### Current status

Enrollment of patients started April 1, 2001. Recently, an interim analysis was performed after the inclusion of 200 patients, having a 30 day follow-up. The Clinical Event Committee recommended continuation of the trial.

## Conclusion

The VIAMI-trial is the first multicenter, randomized, controlled clinical trial, investigating the clinical benefits of percutaneous catheter intervention of the infarct-related artery in patients with demonstrated viability in the early, subacute phase of myocardial infarction.

## Competing interests

The authors declare that they have no competing interests.

## Authors' contribution

RBvL recruited and analysed all data and drafted the manuscript. GV have been involved in drafting the article and revised it critically. OK and JGFB revised the manuscript critically. CAV and FCV have given final approval of the version to be publiced.

## References

[B1] Gunnar RM, Passamani ER, Bourdillon PD, Pitt B, Dixon DW, Rapaport E, Fuster V, Reeves TJ, Karp RB, Russell ROJ, . (1990). Guidelines for the early management of patients with acute myocardial infarction. A report of the American College of Cardiology/American Heart Association Task Force on Assessment of Diagnostic and Therapeutic Cardiovascular Procedures (Subcommittee to Develop Guidelines for the Early Management of Patients with Acute Myocardial Infarction). J Am Coll Cardiol.

[B2] Antman EM, Anbe DT, Armstrong PW, Bates ER, Green LA, Hand M, Hochman JS, Krumholz HM, Kushner FG, Lamas GA, Mullany CJ, Ornato JP, Pearle DL, Sloan MA, Smith SCJ, Alpert JS, Anderson JL, Faxon DP, Fuster V, Gibbons RJ, Gregoratos G, Halperin JL, Hiratzka LF, Hunt SA, Jacobs AK, Ornato JP (2004). ACC/AHA guidelines for the management of patients with ST-elevation myocardial infarction; A report of the American College of Cardiology/American Heart Association Task Force on Practice Guidelines (Committee to Revise the 1999 Guidelines for the Management of patients with acute myocardial infarction). J Am Coll Cardiol.

[B3] Keeley EC, Boura JA, Grines CL (2003). Primary angioplasty versus intravenous thrombolytic therapy for acute myocardial infarction: a quantitative review of 23 randomised trials. Lancet.

[B4] Van de WF, Ardissino D, Betriu A, Cokkinos DV, Falk E, Fox KA, Julian D, Lengyel M, Neumann FJ, Ruzyllo W, Thygesen C, Underwood SR, Vahanian A, Verheugt FW, Wijns W (2003). Management of acute myocardial infarction in patients presenting with ST-segment elevation. The Task Force on the Management of Acute Myocardial Infarction of the European Society of Cardiology. Eur Heart J.

[B5] Hasdai D, Behar S, Wallentin L, Danchin N, Gitt AK, Boersma E, Fioretti PM, Simoons ML, Battler A (2002). A prospective survey of the characteristics, treatments and outcomes of patients with acute coronary syndromes in Europe and the Mediterranean basin; the Euro Heart Survey of Acute Coronary Syndromes (Euro Heart Survey ACS). Eur Heart J.

[B6] Morrow DA, Antman EM, Parsons L, de Lemos JA, Cannon CP, Giugliano RP, McCabe CH, Barron HV, Braunwald E (2001). Application of the TIMI risk score for ST-elevation MI in the National Registry of Myocardial Infarction 3. JAMA.

[B7] (1990). GISSI-2: a factorial randomised trial of alteplase versus streptokinase and heparin versus no heparin among 12,490 patients with acute myocardial infarction. Gruppo Italiano per lo Studio della Sopravvivenza nell'Infarto Miocardico. Lancet.

[B8] (1992). ISIS-3: a randomised comparison of streptokinase vs tissue plasminogen activator vs anistreplase and of aspirin plus heparin vs aspirin alone among 41,299 cases of suspected acute myocardial infarction. ISIS-3 (Third International Study of Infarct Survival) Collaborative Group. Lancet.

[B9] (1993). The effects of tissue plasminogen activator, streptokinase, or both on coronary-artery patency, ventricular function, and survival after acute myocardial infarction. The GUSTO Angiographic Investigators. N Engl J Med.

[B10] Meijer A, Verheugt FW, Werter CJ, Lie KI, van der Pol JM, van Eenige MJ (1993). Aspirin versus coumadin in the prevention of reocclusion and recurrent ischemia after successful thrombolysis: a prospective placebo-controlled angiographic study. Results of the APRICOT Study. Circulation.

[B11] Veen G, Meyer A, Verheugt FW, Werter CJ, de Swart H, Lie KI, van der Pol JM, Michels HR, van Eenige MJ (1993). Culprit lesion morphology and stenosis severity in the prediction of reocclusion after coronary thrombolysis: angiographic results of the APRICOT study. Antithrombotics in the Prevention of Reocclusion in Coronary Thrombolysis. J Am Coll Cardiol.

[B12] Meijer A, Verheugt FW, van Eenige MJ, Werter CJ (1994). Left ventricular function at 3 months after successful thrombolysis. Impact of reocclusion without reinfarction on ejection fraction, regional function, and remodeling. Circulation.

[B13] Nijland F, Kamp O, Verheugt FW, Veen G, Visser CA (1997). Long-term implications of reocclusion on left ventricular size and function after successful thrombolysis for first anterior myocardial infarction. Circulation.

[B14] (1989). Comparison of invasive and conservative strategies after treatment with intravenous tissue plasminogen activator in acute myocardial infarction. Results of the thrombolysis in myocardial infarction (TIMI) phase II trial. The TIMI Study Group. N Engl J Med.

[B15] (1991). SWIFT trial of delayed elective intervention v conservative treatment after thrombolysis with anistreplase in acute myocardial infarction. SWIFT (Should We Intervene Following Thrombolysis?) Trial Study Group. BMJ.

[B16] Erbel R, Pop T, Henrichs KJ, von Olshausen K, Schuster CJ, Rupprecht HJ, Steuernagel C, Meyer J (1986). Percutaneous transluminal coronary angioplasty after thrombolytic therapy: a prospective controlled randomized trial. J Am Coll Cardiol.

[B17] Guerci AD, Gerstenblith G, Brinker JA, Chandra NC, Gottlieb SO, Bahr RD, Weiss JL, Shapiro EP, Flaherty JT, Bush DE, . (1987). A randomized trial of intravenous tissue plasminogen activator for acute myocardial infarction with subsequent randomization to elective coronary angioplasty. N Engl J Med.

[B18] Rogers WJ, Baim DS, Gore JM, Brown BG, Roberts R, Williams DO, Chesebro JH, Babb JD, Sheehan FH, Wackers FJ, . (1990). Comparison of immediate invasive, delayed invasive, and conservative strategies after tissue-type plasminogen activator. Results of the Thrombolysis in Myocardial Infarction (TIMI) Phase II-A trial. Circulation.

[B19] Simoons ML, Arnold AE, Betriu A, de Bono DP, Col J, Dougherty FC, von Essen R, Lambertz H, Lubsen J, Meier B, . (1988). Thrombolysis with tissue plasminogen activator in acute myocardial infarction: no additional benefit from immediate percutaneous coronary angioplasty. Lancet.

[B20] Topol EJ, Califf RM, George BS, Kereiakes DJ, Abbottsmith CW, Candela RJ, Lee KL, Pitt B, Stack RS, O'Neill WW (1987). A randomized trial of immediate versus delayed elective angioplasty after intravenous tissue plasminogen activator in acute myocardial infarction. N Engl J Med.

[B21] Froelicher VF, Perdue S, Pewen W, Risch M (1987). Application of meta-analysis using an electronic spread sheet to exercise testing in patients after myocardial infarction. Am J Med.

[B22] Basu S, Senior R, Raval U, Lahiri A (1997). Superiority of nitrate-enhanced 201Tl over conventional redistribution 201Tl imaging for prognostic evaluation after myocardial infarction and thrombolysis [see comments]. Circulation.

[B23] Bigi R, Desideri A, Bax JJ, Galati A, Coletta C, Fiorentini C, Fioretti PM (2001). Prognostic interaction between viability and residual myocardial ischemia by dobutamine stress echocardiography in patients with acute myocardial infarction and mildly impaired left ventricular function. Am J Cardiol.

[B24] Huitink JM, Visser FC, Bax JJ, van Lingen A, Groenveld AB, Teule GJ, Visser CA (1998). Predictive value of planar 18F-fluorodeoxyglucose imaging for cardiac events in patients after acute myocardial infarction. Am J Cardiol.

[B25] Lee KS, Marwick TH, Cook SA, Go RT, Fix JS, James KB, Sapp SK, MacIntyre WJ, Thomas JD (1994). Prognosis of patients with left ventricular dysfunction, with and without viable myocardium after myocardial infarction. Relative efficacy of medical therapy and revascularization. Circulation.

[B26] Marzullo POVIPWGNCMRIESC, CNR Institute of Clinical Physiology PI (1998). Viability Impact on Prognosis (VIP): Results in 622 patients. J Am Coll Cardiol.

[B27] Nijland F, Kamp O, Verhorst PM, de Voogt WG, Visser CA (2001). In-hospital and long-term prognostic value of viable myocardium detected by dobutamine echocardiography early after acute myocardial infarction and its relation to indicators of left ventricular systolic dysfunction. Am J Cardiol.

[B28] Petretta M, Cuocolo A, Bonaduce D, Nicolai E, Cardei S, Berardino S, Ianniciello A, Apicella C, Bianchi V, Salvatore M (1997). Incremental prognostic value of thallium reinjection after stress- redistribution imaging in patients with previous myocardial infarction and left ventricular dysfunction. J Nucl Med.

[B29] Previtali M, Fetiveau R, Lanzarini L, Cavalotti C, Klersy C (1998). Prognostic value of myocardial viability and ischemia detected by dobutamine stress echocardiography early after acute myocardial infarction treated with thrombolysis. J Am Coll Cardiol.

[B30] Salustri A, Ciavatti M, Seccareccia F, Palamara A (1999). Prediction of cardiac events after uncomplicated acute myocardial infarction by clinical variables and dobutamine stress test. J Am Coll Cardiol.

[B31] Sicari R, Picano E, Landi P, Pingitore A, Bigi R, Coletta C, Heyman J, Casazza F, Previtali M, Mathias WJ, Dodi C, Minardi G, Lowenstein J, Garyfallidis X, Cortigiani L, Morales MA, Raciti M (1997). Prognostic value of dobutamine-atropine stress echocardiography early after acute myocardial infarction. Echo Dobutamine International Cooperative (EDIC) Study. J Am Coll Cardiol.

[B32] Iskander S, Iskandrian AE (1999). Prognostic utility of myocardial viability assessment. Am J Cardiol.

[B33] Carlos ME, Smart SC, Wynsen JC, Sagar KB (1997). Dobutamine stress echocardiography for risk stratification after myocardial infarction [see comments]. Circulation.

[B34] Picano E, Sicari R, Landi P, Cortigiani L, Bigi R, Coletta C, Galati A, Heyman J, Mattioli R, Previtali M, Mathias WJ, Dodi C, Minardi G, Lowenstein J, Seveso G, Pingitore A, Salustri A, Raciti M (1998). Prognostic value of myocardial viability in medically treated patients with global left ventricular dysfunction early after an acute uncomplicated myocardial infarction: a dobutamine stress echocardiographic study. Circulation.

[B35] Samad BA, Frick M, Hojer J, Urstad MJ (1999). Early low-dose dobutamine echocardiography predicts late functional recovery after thrombolyzed acute myocardial infarction. Am Heart J.

[B36] Yoshida K, Gould KL (1993). Quantitative relation of myocardial infarct size and myocardial viability by positron emission tomography to left ventricular ejection fraction and 3-year mortality with and without revascularization. J Am Coll Cardiol.

[B37] Nijland F, Kamp O, Verhorst PM, de Voogt WG, Bosch HG, Visser CA (2002). Myocardial viability: impact on left ventricular dilatation after acute myocardial infarction. Heart.

[B38] Bax JJ, Wijns W, Cornel JH, Visser FC, Boersma E, Fioretti PM (1997). Accuracy of currently available techniques for prediction of functional recovery after revascularization in patients with left ventricular dysfunction due to chronic coronary artery disease: Comparison of pooled data. Journal of the American College of Cardiology.

[B39] (1997). Platelet glycoprotein IIb/IIIa receptor blockade and low-dose heparin during percutaneous coronary revascularization. The EPILOG Investigators. N Engl J Med.

[B40] (1998). Randomised placebo-controlled and balloon-angioplasty-controlled trial to assess safety of coronary stenting with use of platelet glycoprotein-IIb/IIIa blockade. The EPISTENT Investigators. Evaluation of Platelet IIb/IIIa Inhibitor for Stenting. Lancet.

[B41] Montalescot G, Barragan P, Wittenberg O, Ecollan P, Elhadad S, Villain P, Boulenc JM, Morice MC, Maillard L, Pansieri M, Choussat R, Pinton P (2001). Platelet glycoprotein IIb/IIIa inhibition with coronary stenting for acute myocardial infarction. N Engl J Med.

[B42] Mehta SR, Yusuf S, Peters RJ, Bertrand ME, Lewis BS, Natarajan MK, Malmberg K, Rupprecht H, Zhao F, Chrolavicius S, Copland I, Fox KA (2001). Effects of pretreatment with clopidogrel and aspirin followed by long-term therapy in patients undergoing percutaneous coronary intervention: the PCI-CURE study. Lancet.

[B43] Steinhubl SR, Berger PB, Mann JTIII, Fry ET, DeLago A, Wilmer C, Topol EJ (2002). Early and sustained dual oral antiplatelet therapy following percutaneous coronary intervention: a randomized controlled trial. JAMA.

[B44] Rao AK, Pratt C, Berke A, Jaffe A, Ockene I, Schreiber TL, Bell WR, Knatterud G, Robertson TL, Terrin ML (1988). Thrombolysis in Myocardial-Infarction (Timi) Trial - Phase-I - Hemorrhagic Manifestations and Changes in Plasma-Fibrinogen and the Fibrinolytic System in Patients Treated with Recombinant Tissue Plasminogen-Activator and Streptokinase. Journal of the American College of Cardiology.

[B45] Poldermans D, Sozzi FB, Bax JJ, Boersma E, Duncker DJ, Vourvouri E, Elhendy A, Valkema R, Roelandt JR (2001). Influence of continuation of beta blockers during dobutamine stress echocardiography for the assessment of myocardial viability in patients with severe ischemic left ventricular dysfunction. Am J Cardiol.

[B46] Weissman NJ, Levangie MW, Newell JB, Guerrero JL, Weyman AE, Picard MH (1995). Effect of beta-adrenergic receptor blockade on the physiologic response to dobutamine stress echocardiography. Am Heart J.

[B47] Schiller NB, Shah PM, Crawford M, DeMaria A, Devereux R, Feigenbaum H, Gutgesell H, Reichek N, Sahn D, Schnittger I, . (1989). Recommendations for quantitation of the left ventricle by two-dimensional echocardiography. American Society of Echocardiography Committee on Standards, Subcommittee on Quantitation of Two-Dimensional Echocardiograms. J Am Soc Echocardiogr.

